# Bladder cancer with bone marrow metastases and thrombotic microangiopathy: a case report

**DOI:** 10.1093/omcr/omae081

**Published:** 2024-07-30

**Authors:** Khder Yousf, Nagham Daoud, Ali Habib, Rabab Salloum, Firas Hussein

**Affiliations:** Faculty of Medicine, Tishreen University, Lattakia, Syria; Cancer Research Center, Tishreen University, Lattakia, Syria; Faculty of Medicine, Tishreen University, Lattakia, Syria; Cancer Research Center, Tishreen University, Lattakia, Syria; Department of Hematology, Tishreen University Hospital, Lattakia, Syria; Department of Pathology, Tishreen University Hospital, Lattakia, Syria; Department of Hematology, Tishreen University Hospital, Lattakia, Syria

**Keywords:** bladder cancer, thrombotic Microangiopathy (TMA), bone marrow metastases

## Abstract

Bladder cancer is one of the most common cancers of the urinary tract and the 10th most common cancer worldwide according to the World Health Organization (WHO), with a higher incidence in men than in women. Bladder cancer rarely presents with a clinical picture of bone marrow infiltration which may result in thrombotic microangiopathy (TMA). TMA is a syndrome triggered by a wide variety of conditions, some of which are associated with cancer. It is a rare condition in patients with solid tumors, the incidence of which is increasing as awareness of this complication improves. Tumor-induced TMA may exhibit a wide spectrum of clinical manifestations. Here we review the case of a 57-year-old male suffering from transitional bladder cancer with bone marrow infiltration that led to TMA Syndrome. We were able to diagnose the cause and treat the patient in a manner that achieved complete remission of symptoms.

## Introduction

Bladder cancer is one of the most prevalent cancers, with over 430 000 men and women diagnosed worldwide annually [1]. It frequently metastasizes to the lymph nodes, liver, lungs, bone, and adrenal glands, and rarely to the soft tissue and brain [2].

Differences in incidence between different geographical regions exist depending on variables such as smoking, social features, economics and diverse risk factors [3].

The occurrence of cancer-induced thrombotic microangiopathy (TMA) in advanced cancer may result from the invasion of the bone marrow by metastatic tumors. This group of disorders is characterized by ischemic end-organ damage, thrombocytopenia, and microangiopathic hemolytic anemia.

In addition, studies on cancer-induced TMA have suggested that the hemolysis and thrombocytopenia are primarily caused by mechanical obstruction of the vascular lumen by tumor cell emboli [4].

Herein, we report a case of bladder cancer metastatic to the bone marrow that presented as TMA.

## Case presentation

A 57-year-old male smoker with no significant medical history presented to his urologist with a recent onset of painless gross hematuria.

Physical examination revealed bruising, which suggested a coagulation disorder. Based on this, a hematological workup was performed (Table 1).

**Table 1:** Primary hematology workup results

Hemoglobin	5 g/dl
reticulocyte	6.4%
MCV	83,6 fl
WBC count	3.4 *10^3^/mm^3^
platelet count	78 000/μl

**Figure 1:**
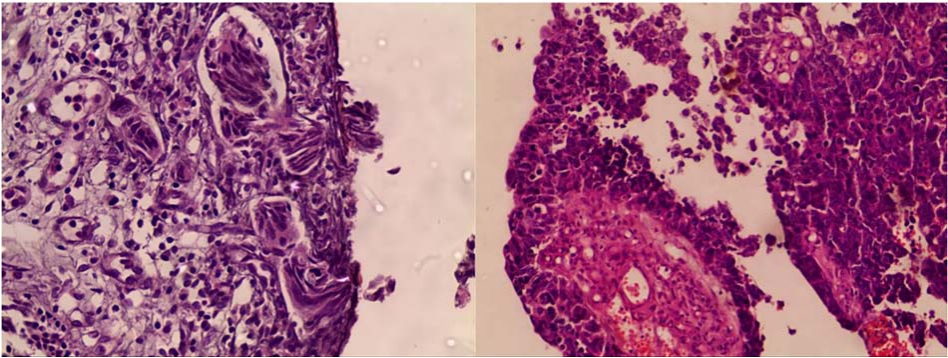
Transurethral resection of bladder tumor pathology findings revealing muscle-invasive bladder cancer with potential for vascular invasion.

The patient was admitted to our hospital’s urology department for further evaluation. Cystoscopy revealed a tumor in the right lateral bladder wall. Transurethral resection of the bladder tumor (TURBT) revealed high-grade urothelial cell carcinoma (grade III–IV) invading the muscularis propria with focal necrosis. Vascular and lymphatic invasion was highly suspected (Fig. 1).

**Figure 2:**
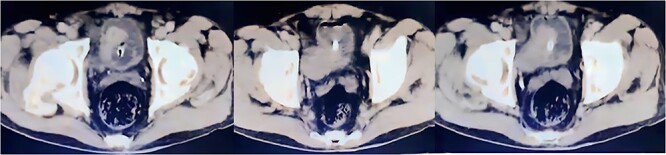
A CT scan of the pelvis reveals the location of bladder cancer.

A full-body CT scan was requested as the pelvic CT showed an enhancing asymmetric formation protruding from the right posterolateral bladder wall, adjacent to the right iliac vessels with no signs of invasion (Fig. 2). The remaining CT scans did not reveal any significant findings or clear evidence of metastatic disease. However, the patient continued to experience bleeding, which resulted in acute anemia. The patient’s condition was complicated by worsening thrombocytopenia. The decision was made to admit the patient to the hematology department. A standard workup for hemolysis was performed (Table 2).

**Table 2:** Standard hematology workup results

LDH	1656 U/L
Bilirubin (Total)	2.6 ml/dl
Bilirubin (Direct)	1.4 ml/dl
Haptoglobin	0.2 g/l
PT	17,4 s
PTT	26 s
Direct anti globulin (Coombs)	Negative

**Figure 3:**
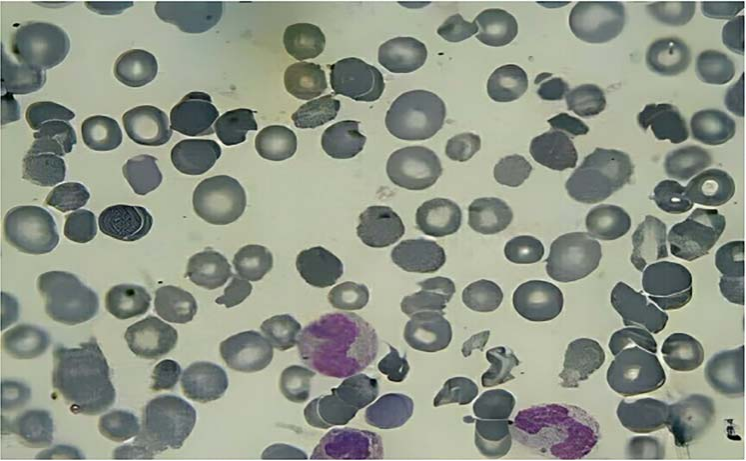
Peripheral blood smear showing the presence of schistocytes, reticulocytes, helmet cell, band neutrophils and unelcleated RBCs.

A peripheral blood smear was made after two days and, demonstrating the presence of schistocytes, reticulocytes, helmet cells, band neutrophils, and unencapsulated RBCs, which established the diagnosis of TMA (Fig. 3). Nevertheless, the patient showed no signs of neurological or renal disorders.

**Figure 4:**
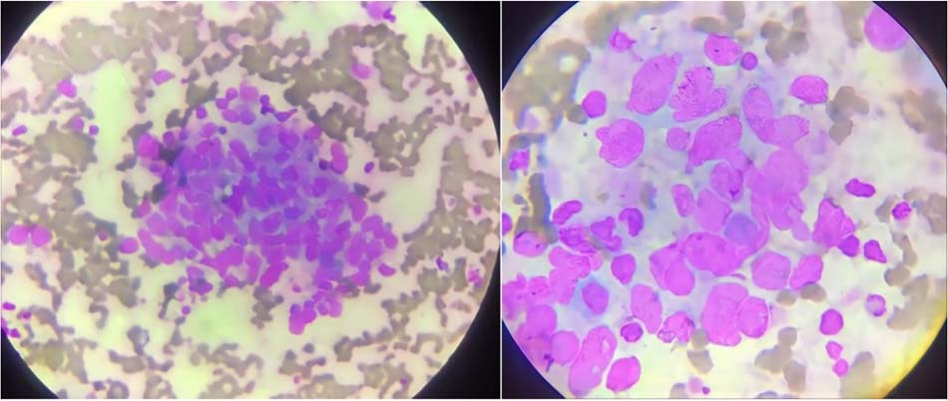
Bone marrow aspiration reveals tumor cell nests.

He was started on plasmapheresis with high-dose steroids for five days. Given the lack of response to previous treatment, a bone marrow aspiration from the iliac crest was performed. The aspiration smear revealed crowded nests of neoplastic cells with large hyperchromatic nuclei, which displayed a picture of metastatic carcinoma (Fig. 4). Owing to these abnormal results, a bone marrow biopsy was performed.

**Figure 5:**
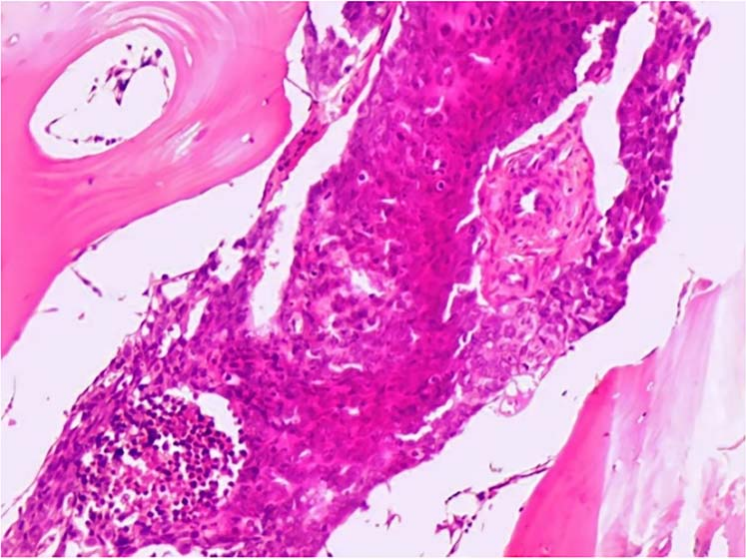
Bone marrow biopsy reveals infiltration with poorly differentiated cells.

Pathological examination of the biopsy specimen revealed that the entire bone marrow was infiltrated by poorly differentiated carcinoma, most likely of urothelial origin, which established the diagnosis of advanced bladder cancer metastatic to the bone marrow (grade IV) (Fig. 5).

The patient was evaluated and considered to be a candidate for platinum–based chemotherapy regimen. Carboplatin was favored over cisplatin due to its lower rate of nephrotoxicity as the patient’s creatinine values increased from 0.6 mg/dl to 1.2 mg/dl after the first dose of cisplatin, and then the patient started on a first-line chemotherapy with gemcitabine on days 1 and 8 and carboplatin on day 8, repeated every 21 days for three cycles. Prophylactic granulocyte colony-stimulating factor (G-CSF) was administered once per cycle, and the results are shown in (Table 3).

**Table 3:** The table shows the stages of improvement of symptoms after each cycle of treatment

After the completion of the first cycle:	The patient had remarkable clinical improvement. In addition, his platelet count started to increase in response to treatment and reached 63.000/μl.
After the second cycle:	The patient’s platelet count increased to 130.000/μl, and his hemoglobin level was 10 g/dl and all other lab results were within normal limits.
At the end of the three—cycle regimen:	The patient was completely stabilized, his platelet count and hemoglobin level went back to normal, and his general condition improved significantly along with his quality of life.

**Figure 6:**
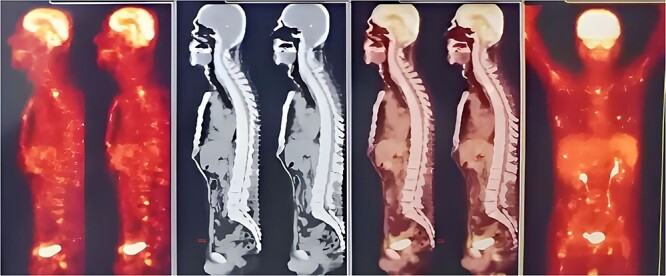
PET Scan detects improvement over treatment.

A new bone scan, which was normal, was requested (Fig. 6). A new peripheral blood smear was obtained. The patient was discharged with periodic follow-up.

## Discussion

Bladder cancer is the most frequent malignancy of the urinary tract [3], with approximately 20% of lesions being muscle—invasive [1]. Muscle—invasive bladder cancer (MIBC) can also progress to metastatic bladder cancer, and metastasizes to the bone marrow [5]. However, bone marrow infiltration by metastatic cancer can result in thrombocytopenia and MAHA. The occurrence of microangiopathic hemolytic anemia and thrombocytopenia in patients can be unexpected and alarming, and these abnormalities are typical clinical features of TMA syndromes [6]. The occurrence of TMA has been documented in a number of cancers, including metastatic gastric cancer, ovarian cancer, prostate cancer, lung cancer, urothelial cancer, lymphoma, myeloproliferative neoplasm, and acute myeloid leukemia [7]. TMA symptoms include renal impairment, neurological impairment, cardiac impairment, hemorrhage, venous thrombosis, and shock [8]. The first step in the diagnostic pathway is the detection of TMA, which is defined as the presence of thrombocytopenia and MAHA. Thrombocytopenia is either absolute (platelet count <150 × 109/L) or relative (>25% reduction in platelet count from baseline). Microangiopathic hemolytic anemia is diagnosed when there are signs of anemia and hemolysis, such as schistocytes in a blood smear, increased lactate dehydrogenase, serum free hemoglobin, reticulocytosis, and diminished haptoglobin which may be a delicate, but nonspecific feature [9]. In our case, there were no signs of neurological or renal disorders, and everything else indicated TMA. The diagnosis was made even though our patient did not have typical symptoms, where urinary bleeding from the bladder manifested by hematuria was the primary symptom. However, the full appearance of symptoms and signs occurs only in a small percentage of patients. Bone marrow aspiration is not required, but may facilitate differential diagnosis, especially in cases of TMA associated with cancer [10]. Plasma exchange therapy (PEX) in combination with steroids is usually the primary treatment, but if clear cases of secondary TMA that are unresponsive to PEX are identified, only supportive care is provided, and further treatment is provided depending on the final diagnosis [8]. In our case, the patient did not show any improvement after treatment with plasma exchange and steroids, which prompted us to treat the cancer with chemotherapy (gemcitabine and carboplatin).

Finally, we achieved complete remission of clinical and laboratory symptoms and signs by using specific chemotherapy for bladder cancer consisting of gemcitabine and carboplatin.

## Conclusion

In this report, we present a rare case of metastatic bladder cancer that metastasized to the bone marrow, leading to secondary TMA syndrome that did not respond to standard treatment with plasma exchange and steroids. We were able to diagnose this condition and completely control the symptoms using chemotherapy with gemcitabine and platinum-based agents.
